# A space division multiplexed free-space-optical communication system that can auto-locate and fully self align with a remote transceiver

**DOI:** 10.1038/s41598-019-55670-1

**Published:** 2019-12-23

**Authors:** Mojtaba Mansour Abadi, Mitchell A. Cox, Rakan E. Alsaigh, Shaun Viola, Andrew Forbes, Martin P. J. Lavery

**Affiliations:** 1 School of Engineering, University of Glasgow, University Avenue, Glasgow, G12 8QQ UK; 2 School of Electrical and Information Engineering, University of the Witwatersrand, Johannesburg, South Africa; 3 School of Physics, University of the Witwatersrand, Johannesburg, South Africa

**Keywords:** Electrical and electronic engineering, Mechanical engineering, Optoelectronic devices and components, Fibre optics and optical communications

## Abstract

Free-Space Optical (FSO) systems offer the ability to distribute high speed digital links into remote and rural communities where terrain, installation cost or infrastructure security pose critical hurdles to deployment. A challenge in any point-to-point FSO system is initiating and maintaining optical alignment from the sender to the receiver. In this paper we propose and demonstrate a low-complexity self-aligning FSO prototype that can completely self-align with no requirement for initial manual positioning and could therefore form the opto-mechanical basis for a mesh network of optical transceivers. The prototype utilises off-the-shelf consumer electrical components and a bespoke alignment algorithm. We demonstrate an eight fibre spatially multiplexed link with a loss of 15 dB over 210 m.

## Introduction

Free-Space Optical (FSO) systems can play a central role in providing high-speed network provision to rural communities and replace fibre network breakages arising from environmental disasters^1^. FSO systems have been widely used in military and space-based communications systems, however the cost of these systems are prohibitive for deployment in rural communities^2,3^. More recently, point-to-point optical systems have been bridging the distance between earth and space, enabling both classical^4^ and quantum^5^ optical systems. Generally, such systems are designed with internal lasers and large area detectors, which limits the ability of these systems to be integrated into existing optical network infrastructure and has limited deployment to bespoke, or ad-hoc networking applications. FSO systems that can seamlessly integrate with standard optical hardware offer many exciting use-cases.

Mechanical tracking systems for maintaining optical alignment in astronomical systems have a long history dating back to the late 1600s, when Giovanni Alfonso Borelli and contemporaries developed the heliostat for tracking a particular point in the night sky^6^. Such technologies are widely implemented into modern telescopes^7^. Laser communication systems, with mechanical alignment and tracking, have been developed based on fast steering-mirror and scanning galvo mirror systems^8^. However, low complexity opto-mechanical systems that can locate and align to any neighbouring transceiver would be an enabler for commercially viable optical mesh networking. Such systems can be used in both urban and data centre environments.

In recent years the deployment of space division multiplexing within FSO systems has been suggested as an exciting method for increasing communication bandwidth over point-to-point links^9–14^. Space division multiplexing systems use orthogonal or spatially separable optical modes to multiplex many encoded data channels within a communication system^10^. Such systems have been demonstrated in both multi-mode fibre^15–17^, in turbulent FSO links^11,18–20^, in submersed channels^21,22^ and combined with retro-reflectors on moving platforms^23^. However, such systems require accurate alignment between sender and receiver without extensive manual pre-alignment or setup; achieving this at a sustainable cost-point has been a limiting factor in the uptake of this technology.

In this paper a low-cost FSO system transceiver that can “out-of-the-box”, locate and self align to any neighbouring transceiver within 1 km is presented. The prototype is based on commercially available consumer components that readily allow for the transition of this system to be available, open sourced, for deployment in low-income or developing nations. The system utilises a telescope mount, which is controlled by a Raspberry Pi 3 (RPi) compact computer, while the communication system makes use of a passively integrated 1 Gbps small form factor pluggable (SFP) transceiver operating at wavelength of 1550 nm. The system utilises a global positioning system (GPS) receiver to identify the location of the transceiver. Once determined, this is communicated to nearby transceivers through a low-speed radio link operating at 446 Mhz. The low-speed radio link supports the communication of the automated alignment instructions between the remote transceivers. A bespoke algorithm has been developed for the transceiver capable of self aligning the system at 200 m in an urban environment with average single mode to few mode coupling losses below 16 dB, adequate for 1 Gbps error free-transmission with widely deployed commercial transceivers. The system can also be used in low-turbulence environments to support plane-wave division multiplexing^12^, where in a data centre environment a 4-channel system with error-free transmission at 1 Gbps per channel is demonstrated.

## Initial location Identification

**Figure 1:**
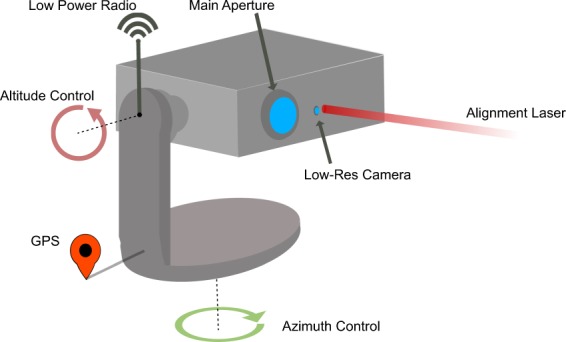
A diagram of the prototype transceiver system that is based on a commercially available motorised Altazimuth mount that has control in both Azimuth and Altitude. To auto-locate, the systems communicate their GPS locations through a low-power radio receiver. Subsequent alignment stages utilise an alignment laser and low-resolution camera to optically align the system. The main aperture collects the light carrying data from another transceiver.

**Figure 2:**
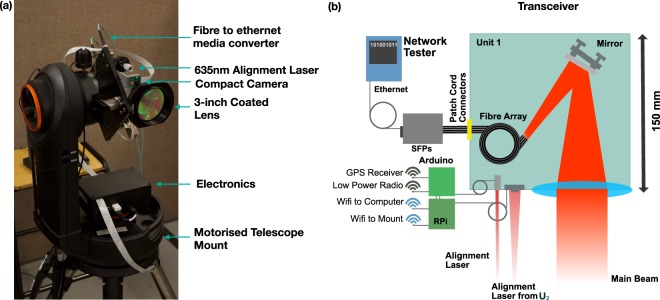
(**a**) The physical system used for the system testing is based on a commercial telescope mount available from Celestron and a range of low-cost off the shelf components. (**b**) A diagram of the system components for one of the two transceiver units.

The transceiver is designed around a motorised Altazimuth mount, commercially available from Celestron (NexStar Evolution) with approximately $5\times {10}^{-6}$ radian step resolution that can be computer controlled. The RPi is used for the system control, and an Arduino is used as the interface device for communicating with the GPS and low-speed radio link operating at 446 Mhz (HiLetgo SI4463; 446 Mhz chosen to match UK handheld radio operational frequency regulations). There are two optical systems within the transceiver. The first is the optical system for managing initial optical alignment comprising a RPi 3 Near IR camera with integrated lens and a 650 nm band pass filter to mitigate stray light from sunlight and environmental background, which is placed off to the side of the main optical collection aperture, see Fig. 1. Alignment is facilitated by using an independent 650 nm laser diode source, slight offset from the camera and mechanically position such that it is approximately parallel to the main optical beam over a test range of 10 m. Second, the main collection system for the optical data signal comprising a 3-inch clear aperture AR coated 200 mm focal length lens, and a fibre array of 50 *μ*m core fibres with 127 *μ*m spacing to provide low complexity spatially separated independent channels Fig. 2(a,b). The fibres within this array are connected to independent SFP transceivers installed within Ethernet media converters to allow for performance testing.

The low-speed low-frequency radio link shares GPS coordinates, latitude (*latA* and *latb* for transmitter, *U*1, and receiver, *U*2, respectively) and longitude (*lonA* and *lonb* for *U*1 and *U*2 respectively), to allow for the initial horizontal and vertical mount orientations to be calculated. The Altazimuth mount requires two variables for controlling alignment, Azimuth (Az), the direction of pointing, and Altitude (Alt), for any height variation between the sender and receiver systems^24,25^. Azimuth is calculated by considering the change in latitude (Δlat) and longitude (Δlon), where

1 $$\begin{array}{rcl}\Delta {\rm{lat}} & = & \mathrm{ln}(\frac{\tan (\frac{{\rm{latB}}}{2}+\frac{\pi }{4})}{\tan (\frac{{\rm{latA}}}{2}+\frac{\pi }{4})})\\ \Delta {\rm{lon}} & = & {\rm{abs}}({\rm{lonA}}-{\rm{lonB}})\end{array}$$

2 $${\rm{Az}}={\rm{atan2}}(\Delta {\rm{lon}},\Delta {\rm{lat}}\mathrm{)}.$$

The altitude angular tilt variable can be calculated by using the altitude information and GPS co-ordinates for the distance, *d*, using the formula

3 $$\begin{array}{rcl}d & = & R\,\arccos [\,\sin ({\rm{latA}})\,\sin ({\rm{latB}})\\  &  & +\,\cos ({\rm{latA}})\,\cos ({\rm{latB}})\,\cos ({\rm{lonA}}-{\rm{lonB}})]\end{array}$$

4 $${\rm{Alt}}={\rm{atan2}}({\rm{AltB}}-{\rm{AltA}},d),$$

where *R* is the radius of the earth. Note that above atan2$(\Delta {\rm{lon}},\Delta {\rm{lat}})$ is used in place of $\arctan (\Delta {\rm{lon}},\Delta {\rm{lat}})$, as arctan does not distinguish between angles that differ by *π*. Unfortunately, the GPS system has an inherent inaccuracy that arises from factors including satellite geometry, atmospheric conditions and other receiver design limitations^26^. The GPS reported position is a centroid of a normal position distribution where the error, *σ*, is defined with a probability of 95%^26^. This positional error region defines the spiral search area (SA) for our mechanical alignment procedure. The horizontal error for our GPS system (MTK3339 GPS chipset) was approximately ${\sigma }_{lat}={\sigma }_{long}=3.6$ m, and an altitude error of approximately ${\sigma }_{alt}=4.5$ m. By combining these errors we define the total radial positional error, ${A}_{s}$, as ${A}_{s}=\sqrt{({\sigma }_{{\rm{lat}}}^{2}+{\sigma }_{{\rm{lat}}}^{2}+{\sigma }_{{\rm{Alt}}}^{2})}=6.8$ m.

## Optical Alignment

**Figure 3:**
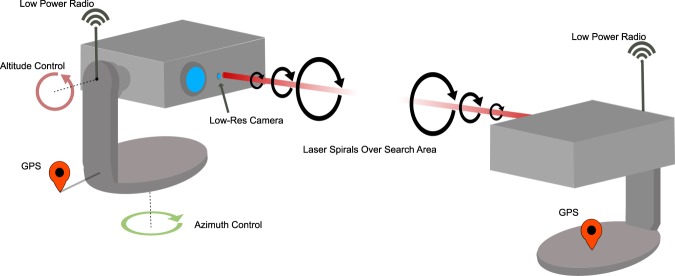
A spiral path is used to physically align the transceivers. The pitch of the spiral is varied based on the alignment stage and distance between transceivers. Such a spiral movement limits the effect of mechanical hysteresis.

**Figure 4:**
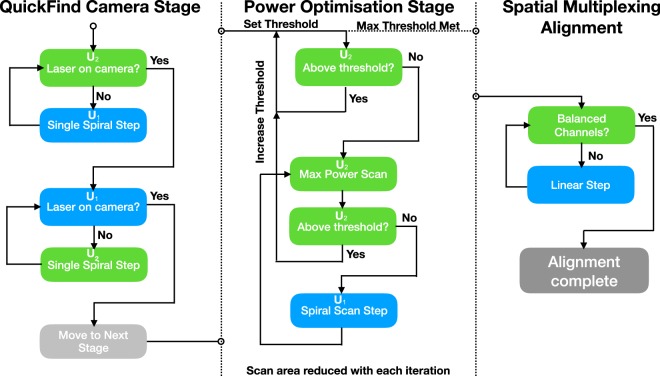
An overview of the key stages completed during optical alignment. A full program diagram for each of the alignment stages is provided in the supplementary information.

Inspired by inter-satellite and ground-to-space optical communications^2,3,27^, our bespoke alignment scheme requires each of the FSO units to perform a spiral search path to locate their partner unit, Fig. 3. From the rough GPS alignment the transmitter (${U}_{1}$) is steered by the computer to points at the receiver (${U}_{2}$), and vice-versa. Using a compact 650 nm laser diode source with a packaged collimating lens as the beacon, and a small CCD camera (Raspberry Pi NOIR Camera v2) the ${U}_{1}$ moves in spiral motion predefined by the GPS accuracy limit. The spiral path has a radial function defined as

5 $$r(\theta )=a{w}_{{\rm{beacon}}}\theta $$

where, *θ* is the azimuthal coordinate in polar form, *a* is a scaling parameter and *w*_beacon_ is the approximate beam waist of the beacon laser at the receiver. Equation 5 is then used to calculate effective latitude and longitude for position at the receiver, where Eqs. 2 and 4 can be used to determine the direction of pointing of the receiver. The camera on ${U}_{2}$ monitors for the beacon light and reports back over the radio link whether it has received any light from the beacon laser. An initial quick scan is performed where ${U}_{1}$ scans for a predetermined amount of time. After this time, ${U}_{2}$ responds with the time at which light was received, and the ${U}_{1}$ then moves back to that position. If no light is detected the ${U}_{1}$ moves along the spiral path for another predefined amount of time, where this time is determined by the hysteresis of the mechanical mount. Once initial optical alignment is determined a second, more accurate, alignment process begins, where ${U}_{1}$ makes a single steps of $a\ast {w}_{{\rm{beacon}}}$, and waits for a response from ${U}_{2}$ on whether it is collecting optical power from the beacon laser. Due to using a small camera aperture, and directional nature of the laser beacon we define a digital region of interest, and intensity threshold value through computational image processing using the OpenCV library installed on the RPi micro-computer. When the intensity reaches the target, ${U}_{2}$ performs a mini spiral scan to locally maximise alignment with the beacon laser and then subsequently communicates with the ${U}_{1}$ to perform a tighter spiral, by reducing parameter $a$, and setting a higher intensity threshold value, see Fig. 4. This process is repeated three times to maximise the positional alignment of the beacon laser with the camera. To prevent saturation, the shutter speed of the camera is changed with each iteration of the alignment stage.

A secondary stage of alignment is carried out to maximise the power coupled specifically into the optical fibre. In this stage of the alignment, both transceivers are required to move sequentially to fully align the beam in both position and tilt. A first power search is carried out, where ${U}_{1}$ moves in a tight spiral pattern and ${U}_{2}$ is continually monitoring power searching of the maximum power position, which repeats with small step size until a threshold power is achieved. Then inspired by the mechanical process of beam walking, the ${U}_{1}$ performs one step along a tight spiral path, where at each step the ${U}_{2}$ scans to maximises the power received at a photo-diode connected to the output of the ${U}_{2}$ fibres.

Finally, as our system is designed to support spatial multiplexing and spatially separated up/down data streams at matched wavelengths, our final stage of alignment assures that channels are directly aligned with of multiple fibres at the transceiver. The laser is switched for the desired channel, and the cross talk determined between any neighbouring channels. If in-balance between channels is detected, the ${U}_{2}$ moves in a linear path, determined by the relative orientation of the multiple fibres with each of the transceivers. To minimise the number of degrees of control we need, it is assumed that the systems are leveled at the both sender and receiver as part of the initial setup of the tripods. If required further rotational alignment could be integrated, however we did not notice any considerable issues with levelling beyond that of the blue’s eye spirit level integrated into the supplied tripod from Celestron.

## Experimental System and Testing

**Figure 5:**
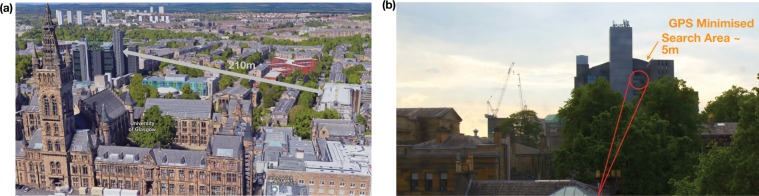
(**a**) Map of the 210 m link across the University of Glasgow Campus with 24 m altitude gain. Imagery © 2019 Google, Maxar Technologies, CNES/Airbus, Landsat/Copernicus, Getmapping plc, Data SIO, NOAA, NGA, GEBCO, IBCAO and U.S Navy. (**b**) A picture from the ${U}_{1}$ looking towards ${U}_{2}$ and outlining the GPS search area.

**Figure 6:**
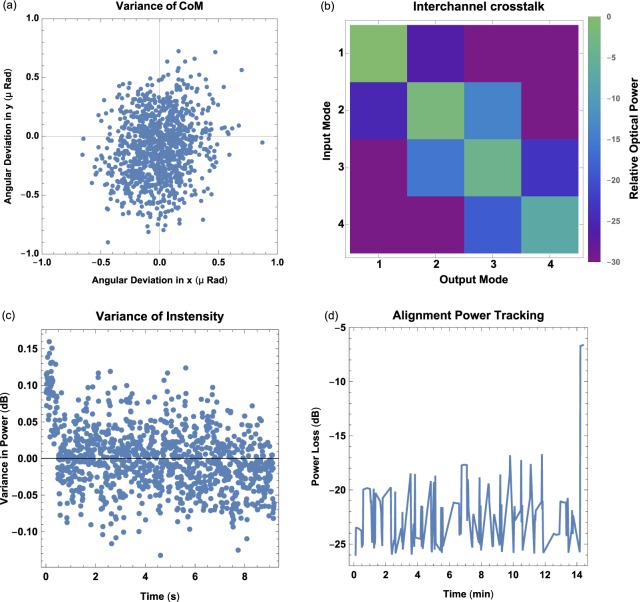
(**a**) Using a high-speed camera the centre of mass of the beam movement at a link distance of 210 m is recorded. (**b**) For a shorter distance of 12.8 m the system apertures are suitable for use with space division multiplexing, where 4 independent channels are demonstrated. (**c**) Variance in intensity after ink distance of 210 m. (**d**) Monitoring the power received into the receiver optical fibre as it performs its auto-alignment over the 12.8 m link.

The system was tested in two link scenarios, first at a distance of approximately 210 m between across the campus of the University of Glasgow, see Fig. 5, and secondly at a link distance of 12.8 m equivalent to a data centre type deployment, Table 1. Over the long distance urban link, both vibrational sensitivity and atmospheric turbulence are critical concerns for the link stability. For the 210 m link the atmospheric turbulence condition during our measurements was determined to have a $D/{r}_{0}=0.565$, where *D* is the aperture size and ${r}_{0}$ is the Fried Parameter, and ${{\rm{C}}}_{n}^{2}=4.483\times 10$^-17^, by computing the seeing disk in the back focal plane of the main optical lens and by monitoring the intensity variance of the source over 8 seconds respectively. The transceivers did not include a high speed centre of mass tracking systems to mitigate these effects, therefore led to increase in the variance of collected optical power over the time of measurement, Fig. 6(c). To analyse the effect of these two affects, a high speed camera (Imaging Source Skyris M618) was placed at the fibre plane in the optical system to measure the centre of mass movement of the received beam. The centre of mass was measured for 8 s at 120 frames per second, where the angular deflection was subsequently calculated based on the optical system. The variation in angle was $0.09\mu$ radians and $0.20769\mu$ radians for tip and tilt respectively, see Fig. 6(a). For the environmental analysis a 850 nm fibre coupled laser source (Thorlabs LPS-830-FC) with 10 dBm of optical optical power was used as 1550 nm sources are not compatible with standard cameras and power meters could be used for the system performance analysis, the main optical lens was changed to an uncoated lens of equivalent focal length to the 1550 nm system. The system is expected to support approximately 5 spatial modes at a range of 210 m and was determined to support spatially separated up and down stream data signals. The relatively small number of available spatial frequencies means higher order turbulence was not measured during our system tests. Larger optical apertures could allow a greater number of spatial mode that one could multiplex, and would lead to more noticeable higher order turbulence that may require the integrating of adaptive optics.

**Table 1:** Coupling Losses and time.

		Urban Link (210 m)	Data Centre Link (15 m)
Power Loss	Average	−16 dB ± 2 dB	−13.8 dB ± 0.1 dB
	Maximum	−15 dB ± 2 dB	−6.6 dB ± 0.1 dB
	Minimum	−21 dB ± 2 dB	−27.7 dB ± 0.1 dB
Time	Average	83 m	12 m 29 s
	Maximum	90 m	28 m 19 s
	Minimum	30 m	1 m 3 s

Shorter systems, operating across streets or in data centre environments can offer access to a larger number of spatial modes without a considerable increase in the aperture size of the system. To analyse the system for use over shorter distances, the system was tested at a range of 12.8 m, Fig. 6(b,d). Both transceivers were equipped with fibres array with eight, 50 *μ*m fibres spaced at 127 *μ*m, to provide low complexity set spatial channels encoded in direction space similar to plane waves, Fig. 2(b)^12^. Alternating fibres were assigned to the up and down stream for the system, allowing for bi-directional performance to be assessed using a commercial performance tester (Ideal Network Unipro Mgig1), certifying a system performance of up to 1 Gbps. We further analysed the channel cross talk between the four spatially separately multiplexed channels showing nearest neighbour crosstalk of -26 dB, indicating the performance is suitable for use with advanced modulation schemes such as Quadrature Amplitude Modulation.

The radio system used to communicate between transceivers and mechanical movement time are the two key parameters that limit alignment time for the system. For each step it takes approximately 50 ms for the mechanical mount to receive the command and execute a movement. To limit this time for performing the initial alignment steps with the beacon laser pointer, a large spot size at the ${U}_{2}$ of around 25 cm is used, allowing an initial quick search to approximately cover the full search area. The hysteresis within the mechanical system alignment leads to a re-positioning error of approximately $6\times {10}^{-4}$ radians. A further mechanical concern is the tracking error from moving the mount at speed, i.e. the error in final position, which was measured for a movement rate of 0.06 radians s^−1^ as $2.6\times {10}^{-3}$ radians and $5\times {10}^{-3}$ radians, for azimuthal and altitude respectively. Therefore, at a distance of 210 m this error can lead to corresponding error in position of 0.55 mm and 1.05 mm. However, moving the system at considerably slower speeds and operating in a closed loop limits the effect and enables precision adequate enough for fibre to fibre alignment.

The overall link latency of the radio connection over the 210 m was measured to be approximately 500 ms. This is because of the low bitrate of the HC-12 transceiver and the limited processing power of the Arduino. A minimum time of one second is required to send the required 16-character message between the transceivers, process the message and then subsequently perform the requested movement of the mount, assuming no packet loss. Potential routes to increase the speed of alignment would be to use a higher frequency and therefore higher bandwidth radio communication module (such as commonly available 2.4 GHz modules which support up to 1 Mbps at lower transmission distances), mounts with lower hysteresis and the ability to dynamically vary the beacon laser pointer size according to the stage of the alignment process.

## Conclusion

In this paper, a system that can auto-locate and fully self align to a remote transceiver over a free-space channel was outlined. This FSO system utilised spatially separated fibres at the back focal plane of the main collection lens to provide SDM channels for increased link capacity. Although a more complex system can perform this task, this paper outlines the process and performance of a system using widely available and cost-effective commercial components. The proposed five-stage method was capable of establishing a high precision, full-duplex FSO link using an image sensor, laser pointer, controller board and some mechanical parts at distances of up to 210 m within ninety minutes. With further optimisation of the radio components of the link, this time could be reduced significantly. We expect our system could be deployed at ranges over 1 Km without considerable increase in alignment time, however, the number of SDM channels would be naturally limited without an increase in the system aperture and alignment precision. Although not as advanced as space-bourne or military systems, the device presented here could be readily replicated for use in developing nations to allow network access to unconnected communities, who are often within a few hundred metres of fibre and are not provided access due to infrastructure constraints^1^. In addition, a portable, self-aligning system would be highly advantageous in providing emergency connectivity in disaster situations where existing fibre infrastructure has been damaged^28,29^.

## Supplementary information

Supplementary Information

## References

[1] Lavery MPJ (2018). Tackling Africa’s digital divide. Nature Photonics.

[2] Baister G, Gatenby PV (1994). Pointing, acquisition and tracking for optical space communications. Electronics & Communication Engineering Journal.

[3] Nguyen, T., Riesing, K., Kingsbury, R. & Cahoy, K. Development of a pointing, acquisition, and tracking system for a CubeSat optical communication module. In *SPIE LASE*, p. 9 (2015).

[4] Boroson, D. M., Biswas, A. & Edwards, B. L. MLCD: overview of NASA’s Mars laser communications demonstration system. *Proc. SPIE 5338, Free-Space Laser Communication Technologies XVI* (2004).

[5] Sheng-Kai Liao (2017). Satellite-to-ground quantum key distribution. Nature.

[6] Middleton WEK (1973). Giovanni Alfonso Borelli and the Invention of the Heliostat. Archive for History of Exact Sciences.

[7] Devaney, N. Review of astronomical adaptive optics systems and plans. *Proc. SPIE 6584, Adaptive Optics for Laser Systems and Other Applications*, 658407 (2007).

[8] https://mynaric.com/.

[9] Richardson DJ, Fini JM, Nelson LE (2013). Space-division multiplexing in optical fibres. Nature Photonics.

[10] Willner AE (2015). Optical communications using orbital angular momentum beams. Advances in Optics and Photonics.

[11] Lavery MPJ (2017). Free-space propagation of high-dimensional structured optical fields in an urban environment. Science Advances.

[12] Lavery MPJ, Huang H, Ren Y, Xie G, Willner AE (2016). Demonstration of a 280 Gbit/s free-space space-division-multiplexing communications link utilizing plane-wave spatial multiplexing. Optics Letters.

[13] Trichili A (2016). Optical communication beyond orbital angular momentum. Scientific Reports.

[14] Amhoud EM, Trichili A, Ooi BS, Alouini M (2019). OAM Mode Selection and Space–Time Coding for Atmospheric Turbulence Mitigation in FSO Communication. Access IEEE.

[15] Huang H (2015). Mode division multiplexing using an orbital angular momentum mode sorter and MIMO-DSP over a graded-index few-mode optical fibre. Scientific Reports.

[16] Leon-Saval SG (2014). Mode-selective photonic lanterns for space-division multiplexing. Optics Express 22.

[17] Arik SO, Askarov D, Kahn JM (2013). Effect of mode coupling on signal processing complexity in mode-division multiplexing. J. Lightwave Technol..

[18] Cox MA (2019). The Resilience of Hermite- and Laguerre-Gaussian Modes in Turbulence. Journal of Lightwave Technology.

[19] Cox MA, Cheng L, Rosales-Guzmán C, Forbes A (2018). Modal Diversity for Robust Free-Space Optical Communications. Physical Review Applied.

[20] Cox MA, Rosales-Guzmán C, Lavery MPJ, Versfeld DJ, Forbes A (2016). On the resilience of scalar and vector vortex modes in turbulence. Optics Express.

[21] Viola, S., Valyrakis, M., Kelly, A. E. & Lavery, M. P. J. Submersed free-space propagation of beams carrying orbital angular momentum. *Proc. SPIE 9991, Advanced Free-Space Optical Communication Techniques and Applications II*, 999103 (2016).

[22] Zhao Y (2017). “Demonstration of data-carrying orbital angular momentum-based underwater wireless optical multicasting link. Optics Express 25.

[23] Li L (2017). High-Capacity Free-Space Optical Communications Between a Ground Transmitter and a Ground Receiver via a UAV Using Multiplexing of Multiple Orbital-Angular-Momentum Beams. Scientific Reports.

[24] Kawase K (2011). A General Formula for Calculating Meridian Arc Length and its Application to Coordinate Conversion in the Gauss-Krüger Projection. Bulletin of the Geospatial Information Authority of Japan.

[25] USA Department of Defense and GPS Navstar Global Positioning System. *Global Positioning System Standard Positioning Service Performance Standard*. 4th edition (2008).

[26] Ranacher, P., Brunauer, R., Trutschnig, W., Van der Spek, S. & Reich, S. Why GPS makes distances bigger than they are. *International Journal of Geographical Information Science* **30**, 316–333, 2016/02/01 (2016). 10.1080/13658816.2015.1086924 PMC4786863 27019610

[27] Kaushal, H., Jain, V. K. & Kar, S. Acuisition, Tracking, and Pointing. In *Free Space Optical Communication* (Springer India, 2017).

[28] Ndagano B, Nape I, Cox MA, Rosales-Guzmán C, Forbes A (2017). “Creation and detection of vector vortex modes for classical and quantum communication. Journal of Lightwave Technology.

[29] Xin, L., Quanyou, S., Jing, M., Siyuan, Y. & Liying, T. Spatial acquisition optimization based on average acquisition time for intersatellite optical communications. In *2010 Academic Symposium on Optoelectronics and Microelectronics Technology and 10th Chinese-Russian Symposium on Laser Physics and Laser Technology Optoelectronics Technology (ASOT)*, pp. 244–248 (2010).

