# Supplementary material for: A space division multiplexed free-space-optical communication system that can auto-locate and fully self align with a remote transceiver
Source: Sci Rep. 2019 Dec 23;9:19687. doi: 10.1038/s41598-019-55670-1 (PMC6928158; doi:10.1038/s41598-019-55670-1)
Supplement: Supplementary file 1 — Supplementary Information [file 41598_2019_55670_MOESM1_ESM.pdf]

Supplemental Information: A space division multiplexed free-space-optical communication system that can auto-locate and fully self align with a remote transceiver

Mojtaba Mansour Abadi[1], Mitchell A. Cox[2], Rakan E. Alsaigh[1], Shaun Viola[1], Andrew Forbes[3] and Martin P.J. Lavery[1]

Below we outline the parameters used within our opto-mechanical systems, Table 2, and a complete programming guide for the approach to allow a reader to replicate the systems for their own application, Figure S1-S5.

Addition Information 1. Detailed alignment code parameters.

Table 2: Code Parameters

Stage	Parameter	Value
Quick search alignment	Number of total iterations	3
	Area above threshold	1%
	a for U_1 main spiral	0.5
	Number of steps	100
Slow search alignment	Number of total iterations	2
	Area above threshold	1.25%
	a for U_1 main spiral	0.5
	Number of steps	5
Half-duplex alignment	Power gain at each iteration	2 dB
	Number of steps	1
	a for U_1 main spiral	0.5
	U_2 power max search area	50 mm
	a for U_2 mini spiral	0.7

Additional Information 2: Detailed alignment code overview.

Outlined is the a detailed overview of the computation process for each of the alignment stages. First stage is the initial GPS alignment of the transceiver units, Figure S1. Second is the two stages of the QuickFind algorithm that perform the initial alignment sweep, Figure S2, and more accurate QuickFind optimisation. Third is the power optimisation stages that perform the alignment for half-duplex, Figure S3 and full-duplex, Figure S4. Finally, the alignment of the separate spatial mode channels used within the system, Figure S5.

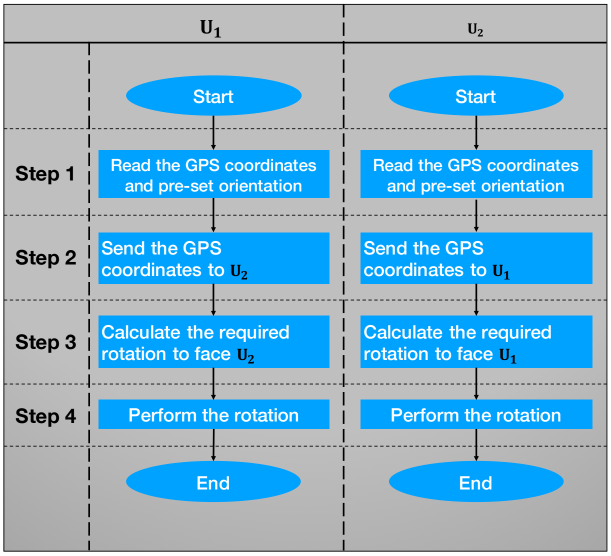

Figure S1. GPS alignment steps for U_1 and U_2.

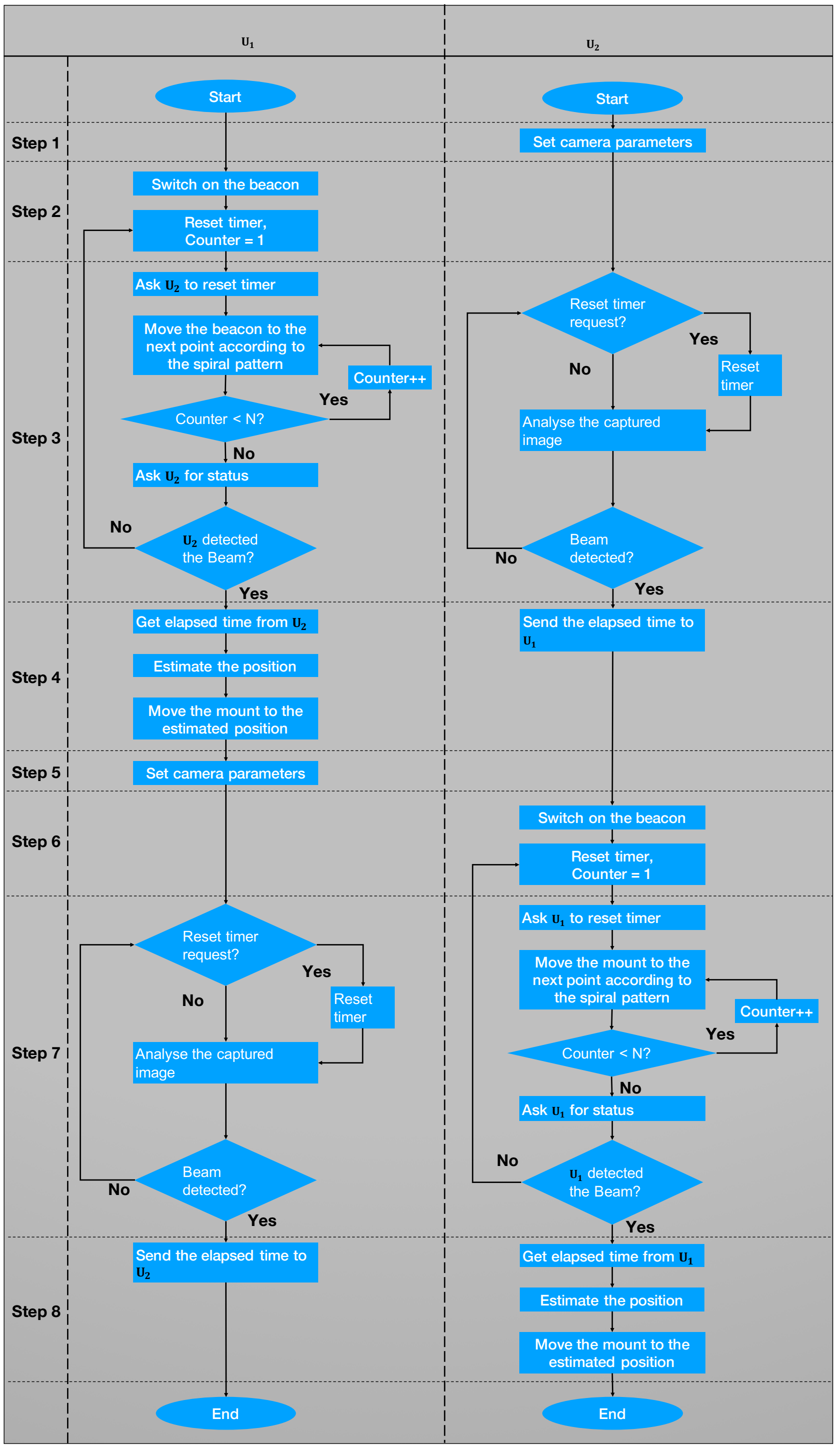

Figure S2. QuickFind initial sweep search alignment steps for U_1 and U_2.

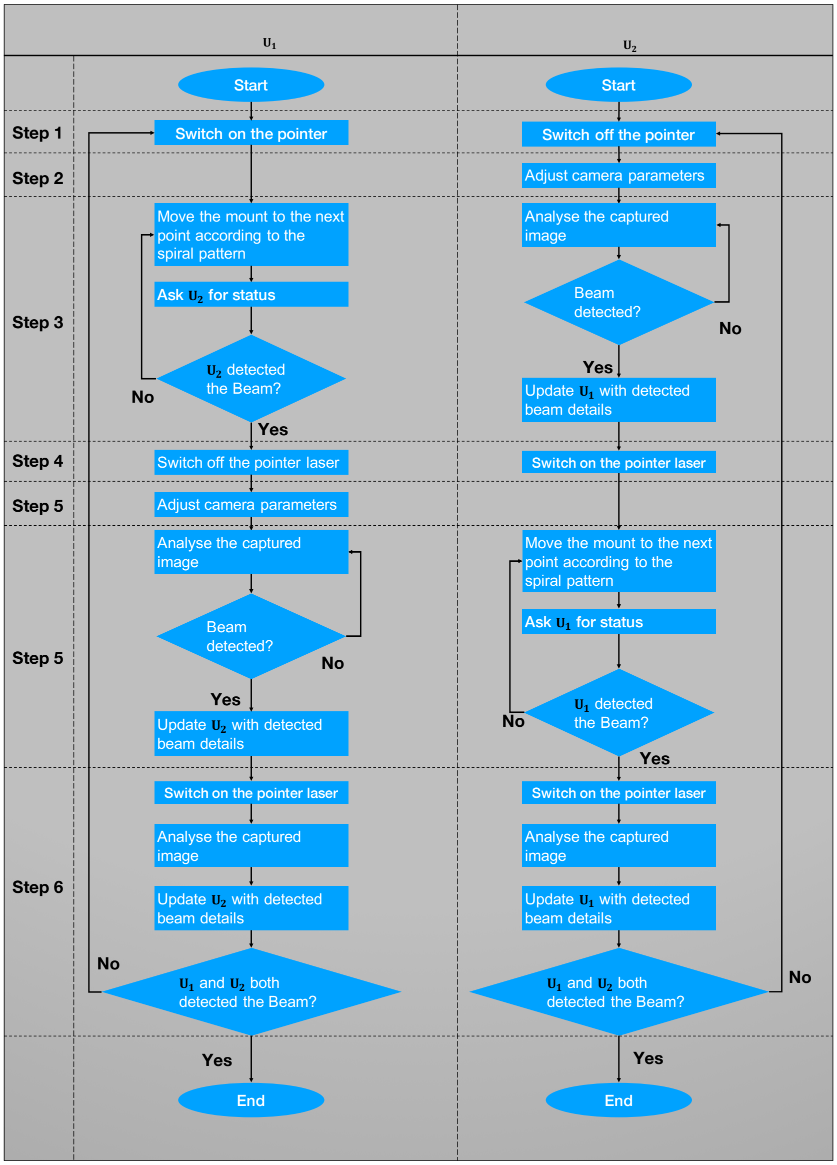

Figure S3. QuickFind optimisation alignment steps for U_1 and U_2.

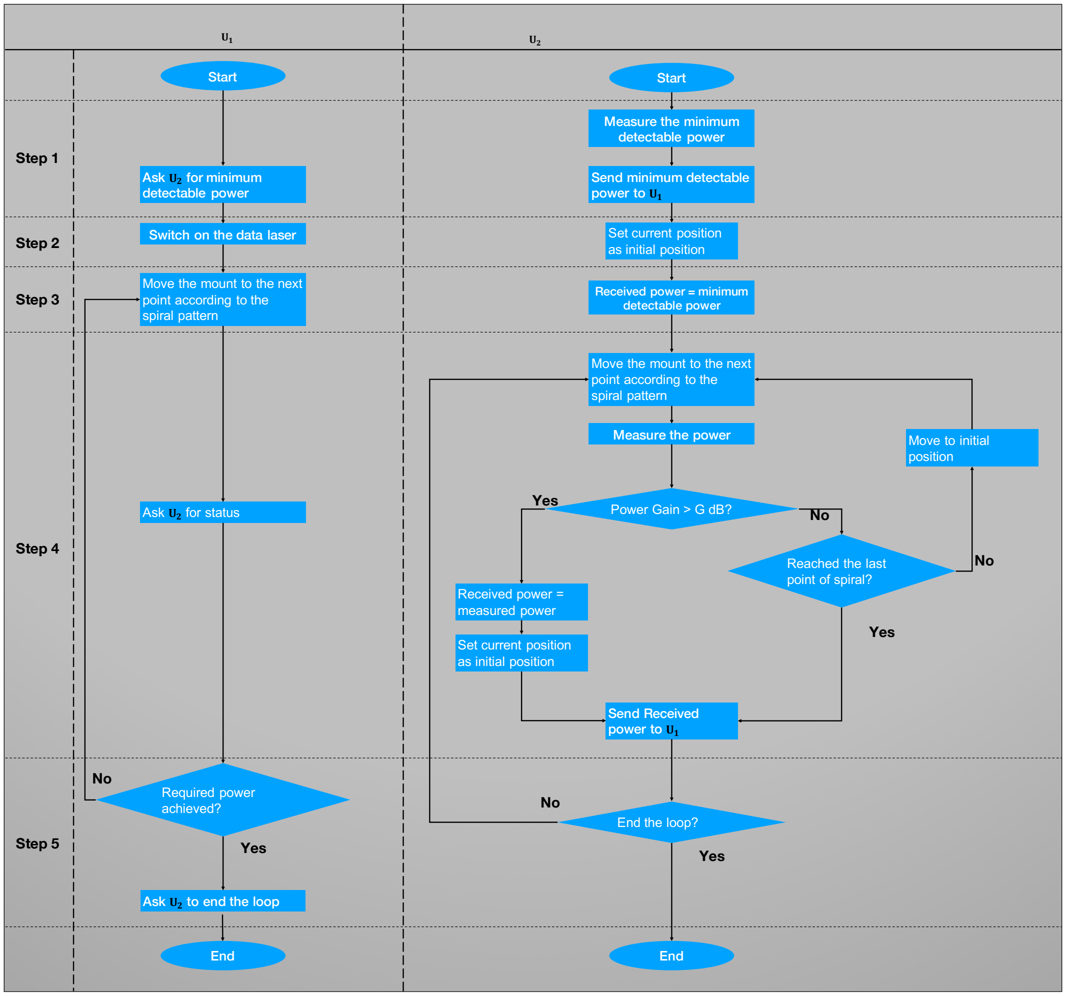

Figure S4. Power Optimisation process for Half-duplex alignment for U_1 and U_2.

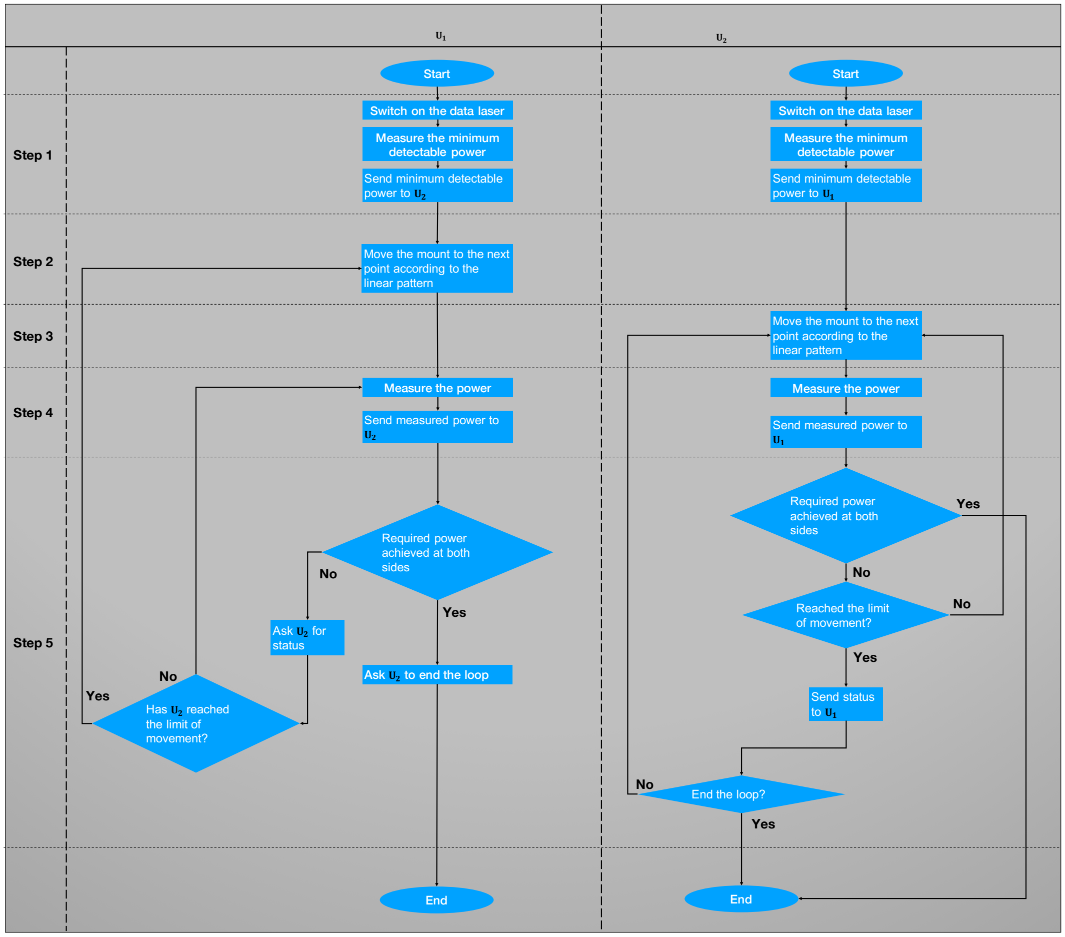

Figure S5. Power Optimisation process for Full-duplex alignment for U_1 and U_2.
